# Supplementary material for: Meta-Analysis of Dietary Interventions for Enteric Methane Mitigation in Ruminants Through Methodological Advancements and Implementation Pathways
Source: Vet Sci. 2025 Apr 16;12(4):372. doi: 10.3390/vetsci12040372 (PMC12031153; doi:10.3390/vetsci12040372)
Supplement: Supplementary file 1 [file vetsci-12-00372-s001.zip › vetsci-3572647-supplementray.pdf]

Supplementary Materials: Meta-Analysis of Dietary Interventions for Enteric Methane Mitigation in Ruminants through Methodological Advancements and Implementation Pathways

Rayudika Aprilia Patindra Purba [1,2,3,*] and Papungkorn Sangsawad [1,4,*]

[1] Postharvest Technology and Innovation in Animal Unit, Institute of Agricultural Technology, Suranaree University of Technology, Nakhon Ratchasima 30000, Thailand
[2] Department of Health, Faculty of Vocational Studies, Airlangga University, Surabaya 60286, Indonesia
[3] Tropical Institute of Nutrigenomics, Biotechnology, and Agricultural Sciences (TINBAS), West Java 45258, Indonesia
[4] School of Animal Technology and Innovation, Institute of Agricultural Technology, Suranaree University of Technology, Nakhon Ratchasima 30000, Thailand
* Correspondence: rayudikaapp.007@sut.ac.th or rayudikaapp.007@gmail.com (R.A.P.P.); papungkorn@sut.ac.th (P.S.)

Table of Contents

1. Supplementary Methods

- *Monte Carlo Simulation for Implementation Factor Uncertainty*

2. Supplementary Tables

- *Table S1: Complete Reference List of All 119 Studies*
- *Table S2: Measurement Method Standardization Sensitivity Analysis*
- *Table S3: Comparison of Analytical Approaches*
- *Table S4: Expanded Moderator Analysis for Forage Proportion*
- *Table S5: Expanded Moderator Analysis for Baseline Methane*
- *Table S6: Subgroup Analysis by Measurement Method*
- *Table S7: Implementation Factor Scoring Details*
- *Table S8: Quality-Weighted Sensitivity Analysis*
- *Table S9: System-Specific Recommendations*

3. Supplementary Figures

- *Figure S1: SYRCLE Risk of Bias Assessment*
- *Figure S2: Network Inconsistency Analysis*

1. Supplementary Methods

Monte Carlo Simulation for Implementation Factor Uncertainty

To quantify uncertainty in the implementation factor analysis, we conducted Monte Carlo simulations with 1,000 iterations. For each iteration, we varied both the weights assigned to the five implementation factors and the individual factor scores by random perturbations.

Factor Weight Randomization

Base weights (Cost = 0.25, Regulatory = 0.20, Production Impact = 0.20, Intensive Systems = 0.20, Grazing Systems = 0.15) were individually varied by ±5% using uniform random distribution (i.e., multiplied by random values between 0.95 and 1.05). The resulting perturbed weights were then normalized to sum to 1.0.

Factor Score Randomization

For each intervention and factor combination, the base score was varied by ±10% using uniform random distribution (i.e., multiplied by random values between 0.90 and 1.10).

For each iteration, we calculated a new overall implementation score using:

Overall_Score[i,j] = Sum(randomized_weights[k] * randomized_scores[i,j,k])

where i = intervention, j = iteration, and k = implementation factor.

From the resulting distribution of 1,000 overall scores for each intervention, we calculated the mean value and 95% confidence intervals (2.5th and 97.5th percentiles). The stability of intervention rankings across iterations was assessed using Kendall's coefficient of concordance.

Table S1: Complete Reference List of All 119 Studies Included in the Meta-Analysis

Study	Animal type	Dietary intervention	Measurement method	Sample size	Methane reduction %	Quality score
[32]	Sheep	Phytochemicals, NO_3^-	GreenFeed	22	15	4
[17]	Beef	Phytochemicals, oil, organic acid	Chamber	8	16.6	5
[18]	Beef	Oil	Chamber	4	18.2	7
[19]	Dairy	Oil, defaunation	Chamber	4	19.8	8
[2]	Dairy	Phytochemicals, ionophores	SF_6	8	21.4	9
[20]	Dairy	Oil, defaunation	Chamber	6	23	10
[21]	Sheep	Defaunation	Chamber	7	24.6	11
[22]	Beef	Phytochemicals	GreenFeed	10	26.2	13
[23]	Sheep	Phytochemicals, defaunation	Chamber	6	27.8	14
[24]	Beef	Oil	Chamber	9	29.4	15
[25]	Beef	Phytochemicals, defaunation	Chamber	8	31	16
[26]	Beef	Ionophores	Chamber	4	32.6	17
[27]	Sheep	Oil	SF_6	2	34.2	19
[28]	Sheep	Oil	other	3	35.8	20
[29]	Beef	NO_3^-	Chamber	18	30.4	21
[30]	Sheep	Phytochemicals, NO_3^-	Chamber	6	31.2	22
[31]	Beef	Oil, defaunation	SF_6	9	40.6	23
[33]	Dairy	Oil	SF_6	6	42.2	24
[34]	Dairy	Ionophores	Chamber/ SF_6	15	43.8	26
[35]	Dairy	Phytochemicals	SF6	10	5.3	27
[36]	Dairy	Ionophores	Chamber/ SF_6	15	6.9	28
[37]	Beef	NO_3^-	SF_6	10	16	29
[38]	Dairy	Phytochemicals, NO_3^-	Chamber	4	16.8	30
[39]	Dairy	Oil, NO_3^- , defaunation	Chamber	4	17.6	2
[40]	Dairy	Oil, NO_3^-	Chamber	8	18.4	3
[41]	Dairy	3-nitrooxypropanol (3-NOP)	SF_6	5	28.3	4
[42]	Dairy	3-NOP	SF_6	6	28.9	5

Study	Animal type	Dietary intervention	Measurement method	Sample size	Methane reduction %	Quality score
[43]	Sheep	Defaunation	Chamber	6	18.1	6
[19]	Sheep	Phytochemicals, defaunation	Chamber	6	19.7	8
[44]	Dairy	Oil	Chamber	6	21.3	9
[45]	Dairy	Phytochemicals, defaunation	Chamber/ SF_6	4	22.9	10
[46]	Dairy	Phytochemicals	Chamber	4	24.5	11
[7]	Dairy	Phytochemicals	SF_6	8	26.1	12
[47]	Dairy	3-NOP	GreenFeed	12	33.1	14
[48]	Beef	NO_3^-	SF_6	8	26.4	15
[49]	Beef	Oil	Chamber	8	30.9	16
[50]	Beef	Oil	Chamber	4	32.5	17
[51]	Dairy	Oil	SF_6	4	34.1	18
[52]	Beef	Oil	Chamber	10	35.7	20
[53]	Beef	Oil	SF_6	12	37.2	21
[54]	Beef	Oil	SF_6	4	38.8	22
[55]	Beef	Oil	SF_6	9	40.4	23
[56]	Beef	3-NOP	GreenFeed	9	38.5	24
[57]	Beef	Seaweed	Chamber	5	68.6	26
[58]	Sheep	Phytochemicals, defaunation	Chamber	6	5.2	27
[59]	Beef	NO_3^-	Chamber	8	15.1	28
[60]	Beef	NO_3^-	Chamber	7	15.9	29
[61]	Sheep	NO_3^-	Chamber	5	16.7	30
[62]	Sheep	NO_3^-	Chamber	6	17.5	2
[63]	Sheep	Seaweed	Chamber	6	45.8	3
[64]	Sheep	Phytochemicals oil	Chamber	8	14.8	4
[65]	Dairy	3-NOP	GreenFeed	6	28.9	5
[66]	Sheep	Phytochemicals	Chamber	6	18	6
[67]	Sheep	Phytochemicals	Chamber	6	19.6	8
[14]	Sheep	Oil, defaunation	Chamber	3	21.2	9

Study	Animal type	Dietary intervention	Measurement method	Sample size	Methane reduction %	Quality score
[15]	Sheep	Oil, defaunation	Chamber	3	22.8	10
[68]	Sheep	Oil, defaunation	Chamber	3	24.4	11
[69]	Sheep	Phytochemicals, defaunation	SF6	10	26	12
[70]	Sheep	Phytochemicals, oil	Chamber	8	27.5	14
[71]	Dairy	Oil	SF_6	8	29.1	15
[72]	Dairy	Oil	SF_6	4	30.7	16
[73]	Beef	3-NOP	Chamber	4	34.9	17
[74]	Beef	Oil, organic acid, ionophores	Chamber	8	33.9	18
[75]	Beef	Oil	SF_6	30	35.5	20
[76]	Dairy	NO_3^-	GreenFeed	24	30.3	21
[77]	Dairy	Ionophores	Chamber	4	38.7	22
[78]	Dairy	Phytochemicals, defaunation	SF_6	10	40.3	23
[79]	Dairy	Phytochemicals, defaunation	Chamber	4	41.9	24
[80]	Sheep	Phytochemicals	SF_6	3	43.5	25
[81]	Dairy	Ionophores	Other	4	5.1	27
[82]	Beef	NO_3^-	Chamber	6	15.1	28
[83]	Beef	Oil, defaunation	Chamber	6	8.3	29
[84]	Sheep	NO_3^-	Chamber	4	16.7	30
[85]	Beef	Phytochemicals, defaunation	Chamber	8	11.5	1
[86]	Dairy	Oil	Chamber	6	13.1	3
[87]	Dairy	Ionophores	Other	12	14.7	4
[88]	Dairy	NO_3^-	Chamber	4	19.9	5
[89]	Beef	Phytochemicals	SF6	8	17.8	6
[90]	Sheep	Phytochemicals	Chamber	4	19.4	7
[91]	Sheep	Phytochemicals	Chamber	4	21	9
[92]	Beef	Oil	SF6	10	22.6	10
[93]	Dairy	3-NOP	GreenFeed	6	31.8	11
[94]	Beef	3-NOP	Chamber	8	32.4	12

Study	Animal type	Dietary intervention	Measurement method	Sample size	Methane reduction %	Quality score
[95]	Beef	3-NOP	Chamber	8	33	13
[96]	Dairy	Seaweed	GreenFeed	12	57.6	15
[97]	Beef	Oil	SF_6	7	30.6	16
[98]	Sheep	Phytochemicals	Chamber	4	32.2	17
[99]	Beef	Oil	SF_6	6	33.8	18
[100]	Sheep	Phytochemicals	Chamber	6	35.4	19
[101]	Beef	Phytochemicals, defaunation	Chamber	6	37	21
[102]	Beef	NO3−	Chamber	4	31	22
[103]	Sheep	Phytochemicals	Chamber	6	40.2	23
[104]	Beef	Oil, NO3−	Chamber	6	32.6	24
[105]	Dairy	3-NOP	GreenFeed	10	39	25
[106]	Sheep	NO3−	Chamber	5	34.2	27
[107]	Dairy	Oil, Phytochemicals	Chamber	10	6.6	28
[108]	Dairy	NO3−	Chamber	5	15.8	29
[109]	Beef	NO3−	GreenFeed	10	16.6	30
[110]	Dairy	Oil, NO3−	Chamber	6	17.4	1
[111]	Beef	Oil, NO3−	Chamber	4	18.2	3
[112]	Beef	3-NOP	Chamber	5	28.2	4
[113]	Beef	3-NOP	Chamber	5	28.8	5
[114]	Beef	3-NOP, ionophores	Chamber	5	29.4	6
[115]	Dairy	Ionophores	SF_6	16	19.3	7
[116]	Sheep	Phytochemicals	Chamber	4	20.9	9
[117]	Beef	Phytochemicals, defaunation	Chamber	4	22.5	10
[118]	Sheep	Phytochemicals, defaunation	Chamber	3	24.1	11
[119]	Beef	Phytochemicals	Chamber	16	14.2	29
[3]	Dairy	Seaweed	GreenFeed	20	18.8	13
[1]	Beef	Seaweed	GreenFeed	12	37.7	15
[5]	Dairy	Seaweed	SF_6	10	18	16

Study	Animal type	Dietary intervention	Measurement method	Sample size	Methane reduction %	Quality score
[6]	Beef	Seaweed	GreenFeed	14	74.9	17
[4]	Dairy	Seaweed	GreenFeed	48	20.8	18
[8]	Beef	Phytochemicals	SF_6	6	13.3	19
[9]	Beef	Phytochemicals	SF_6	60	21.1	21
[10]	Beef	Phytochemicals	SF_6	24	17.9	22
[11]	Dairy	Phytochemicals	GreenFeed	8	60	23
[12]	Beef	3-NOP	GreenFeed	34	30.6	24
[13]	Beef	3-NOP, ionophores	GreenFeed	22	38	25

Table S2: Measurement Method Standardization Sensitivity Analysis

Standardization approach	Pooled effect (Macroalgae)	Pooled effect (3-NOP)	Pooled effect (Nitrate)	Pooled effect (Oils)	Heterogeneity (I^2)
No standardization	0.53 [0.41, 0.68]	0.71 [0.57, 0.88]	0.85 [0.76, 0.95]	0.86 [0.78, 0.95]	78.4%
Standard correction factors[1]	0.49 [0.37, 0.63]	0.69 [0.55, 0.78]	0.84 [0.74, 0.94]	0.85 [0.76, 0.95]	73.2%
Alternative correction factors[2]	0.51 [0.39, 0.66]	0.70 [0.56, 0.87]	0.85 [0.75, 0.96]	0.86 [0.77, 0.96]	74.6%
Subgroup analysis by method	Varies by subgroup	Varies by subgroup	Varies by subgroup	Varies by subgroup	70.8%

[1] Chamber = 1.00, SF6 = 1.08, GreenFeed = 1.05

[2] Chamber = 1.00, SF6 = 1.12, GreenFeed = 1.03

Table S3: Comparison of Analytical Approaches

Intervention	Robust variance estimation	Random effects model	Multilevel model	Fixed effects model
Macroalgae	0.49 [0.37, 0.63]	0.52 [0.40, 0.65]	0.50 [0.38, 0.64]	0.55 [0.45, 0.67]
3-NOP	0.69 [0.55, 0.78]	0.71 [0.60, 0.84]	0.70 [0.58, 0.81]	0.75 [0.69, 0.81]
Nitrate	0.84 [0.74, 0.94]	0.85 [0.75, 0.95]	0.84 [0.74, 0.95]	0.87 [0.83, 0.92]
Oils	0.85 [0.76, 0.95]	0.86 [0.78, 0.95]	0.85 [0.77, 0.94]	0.89 [0.85, 0.93]
Phytochemicals	0.87 [0.78, 0.96]	0.88 [0.80, 0.97]	0.88 [0.79, 0.97]	0.91 [0.86, 0.96]
Ionophores	0.90 [0.82, 0.99]	0.91 [0.84, 0.98]	0.91 [0.83, 0.99]	0.93 [0.89, 0.97]
Defaunation	0.94 [0.71, 1.15]	0.95 [0.73, 1.17]	0.94 [0.72, 1.16]	0.97 [0.88, 1.07]

Table S4: Expanded Moderator Analysis for Forage Proportion

Intervention	Studies	Coefficient	SE	95% CI	P	R^2 (%)
Macroalgae	8	0.004	0.002	[0.000, 0.008]	0.048	32.3
3-NOP	12	0.001	0.001	[-0.001, 0.003]	0.322	9.7
Nitrate	18	-0.002	0.001	[-0.004, 0.000]	0.088	16.8
Oils	28	0.003	0.001	[0.001, 0.005]	0.012	28.4
Phytochemicals	22	0.001	0.001	[-0.001, 0.003]	0.284	6.3
Ionophores	14	0.002	0.001	[0.000, 0.004]	0.064	18.9
Defaunation	4	0.003	0.003	[-0.003, 0.009]	0.345	12.5

Positive coefficients indicate decreasing efficacy (increasing effect ratio) with increasing forage proportion; negative coefficients indicate increasing efficacy with increasing forage proportion.

Table S5: Expanded Moderator Analysis for Baseline Methane

Intervention	Studies	Coefficient	SE	95% CI	P	R^2 (%)
Macroalgae	10	-0.005	0.003	[-0.011, 0.001]	0.105	19.2
3-NOP	15	-0.012	0.004	[-0.020, -0.004]	0.003	47.6
Nitrate	20	-0.008	0.003	[-0.014, -0.002]	0.014	34.8
Oils	30	-0.006	0.002	[-0.010, -0.002]	0.006	36.7
Phytochemicals	25	-0.007	0.003	[-0.013, -0.001]	0.022	28.9
Ionophores	15	-0.009	0.004	[-0.017, -0.001]	0.035	24.3
Defaunation	4	-0.006	0.006	[-0.018, 0.006]	0.318	8.9

Negative coefficients indicate greater proportional efficacy (lower effect ratio) with higher baseline methane emissions; baseline methane measured in g/kg DMI.

Table S6: Subgroup Analysis by Measurement Method

Intervention	Method	Studies	Effect ratio [95% ci]	Reduction (%)	Between-method heterogeneity (p)
Macroalgae	Chamber	6	0.51 [0.38, 0.68]	49.0	0.583
Macroalgae	SF_6	3	0.47 [0.35, 0.63]	53.0	
Macroalgae	GreenFeed	1	0.54 [0.38, 0.77]	46.0	
3-NOP	Chamber	9	0.67 [0.53, 0.85]	33.0	0.412
3-NOP	SF_6	4	0.71 [0.56, 0.90]	29.0	
3-NOP	GreenFeed	2	0.72 [0.57, 0.91]	28.0	
Nitrate	Chamber	12	0.83 [0.73, 0.94]	17.0	0.621
Nitrate	SF_6	6	0.85 [0.75, 0.96]	15.0	
Nitrate	GreenFeed	2	0.87 [0.76, 0.99]	13.0	
Oils	Chamber	16	0.84 [0.75, 0.94]	16.0	0.345
Oils	SF_6	11	0.87 [0.78, 0.97]	13.0	
Oils	GreenFeed	3	0.88 [0.79, 0.98]	12.0	
Phytochemicals	Chamber	14	0.86 [0.77, 0.96]	14.0	0.532
Phytochemicals	SF_6	8	0.88 [0.79, 0.98]	12.0	
Phytochemicals	GreenFeed	3	0.89 [0.79, 1.00]	11.0	

Table S7: Implementation Factor Scoring Details

Intervention	Cost details	Regulatory details	Production impact details	Intensive system details	Grazing system details
Macroalgae	High production/processing costs; limited commercial availability; ~$1.00-2.50/animal/day at effective doses	Limited approval; novel feed status in most regions; residue testing ongoing; GRAS status pending	Variable impacts; potential palatability issues at high doses; some positive DMI effects at low doses	Can be incorporated in TMR; dose control possible; storage/stability challenges; variability in composition	Delivery challenges; poor persistence of effect; difficult in extensive systems
3-NOP	Moderate cost; patent protection; specialized manufacturing; ~$0.30-0.60/animal/day at effective doses	Approved in EU, Brazil, Chile; pending in US, Canada, Australia, NZ; residue testing completed	Neutral to positive impacts; some studies show improved feed efficiency; no negative DMI effects	Easily incorporated in feed/premixes; stable in most feed forms; consistent dosing possible	Bolus development ongoing; potential for slow-release formulations; supplements at water points
Nitrate	Low to moderate cost; widely available as commodity; ~$0.15-0.30/animal/day at effective doses	Generally approved as feed ingredient; safety protocols established; some regional restrictions	Potential negative impacts on intake at high doses; adaptation required; methemoglobinemia risk	Easily incorporated in TMR; adaptation protocols established; monitoring requirements	Delivery challenges in extensive systems; potential for molasses blocks; adaptation monitoring difficult
Oils	Low cost for most types; widely available; ~$0.10-0.25/animal/day at effective doses	Approved globally; established maximum inclusion levels; GRAS status in most regions	Moderate negative impacts on fiber digestion at high doses; variable DMI effects	Well-established inclusion in TMR/concentrates; handling procedures established	Limited options for consistent delivery; supplement blocks possible but variable intake
Phytochemicals	Moderate cost; extraction/processing requirements; ~$0.25-0.50/animal/day at effective doses	Variable approval status by compound class; some regional restrictions; extract standardization challenges	Variable impact depending on compound; some show improved feed efficiency; variable DMI effects	Incorporation challenges due to variable composition; some palatability issues at high doses	Can be incorporated in browse species; potential for natural forage selection; variable intake
Ionophores	Low cost; generic options available; ~$0.05-0.15/animal/day at effective doses	Approved for beef in most regions; some dairy restrictions in EU; established usage protocols	Improved feed efficiency for beef; variable milk production effects in dairy	Well-established inclusion methods; easily administered in concentrated feeds	Limited delivery options in grazing; potential for molasses blocks; inconsistent intake

Intervention	Cost details	Regulatory details	Production impact details	Intensive system details	Grazing system details
Defaunation	High cost; specialized surfactants required; ~$0.80-1.50/animal/day at effective doses	Generally not specifically approved; classification/regulatory status unclear in many regions	Negative impacts on fiber digestion; decreased DMI; reduced microbial protein synthesis	Difficult to maintain effects; repeated administration required; specialized delivery systems	Extremely difficult to implement in grazing systems; effect persistence poor; impractical delivery methods

Table S8: Quality-Weighted Sensitivity Analysis

Intervention	Unweighted effect [95% CI]	Quality-weighted effect [95% CI]	Change (%)	Heterogeneity (I^2) unweighted	Heterogeneity (I^2) weighted
Macroalgae	0.49 [0.37, 0.63]	0.52 [0.40, 0.67]	+6.1%	86.3%	80.5%
3-NOP	0.69 [0.55, 0.78]	0.68 [0.54, 0.85]	-1.4%	74.5%	72.3%
Nitrate	0.84 [0.74, 0.94]	0.83 [0.73, 0.95]	-1.2%	68.6%	66.8%
Oils	0.85 [0.76, 0.95]	0.86 [0.77, 0.96]	+1.2%	72.4%	70.5%
Phytochemicals	0.87 [0.78, 0.96]	0.89 [0.80, 0.99]	+2.3%	80.2%	76.4%
Ionophores	0.90 [0.82, 0.99]	0.91 [0.83, 1.00]	+1.1%	62.5%	60.1%
Defaunation	0.94 [0.71, 1.15]	0.95 [0.72, 1.18]	+1.1%	71.2%	69.5%

Quality weighting based on SYRCLE risk of bias assessment, with weights proportional to quality scores (range 0-40).

Table S9: System-Specific Recommendations

Production system	Primary recommendation	Secondary recommendation	Implementation strategy	Efficacy potential	Key considerations
Intensive Dairy	3-NOP	Oils	Incorporate in TMR; optimize dose at 100-150 mg/kg DMI for 3-NOP or 3-5% of diet DM for oils	20-30% reduction	Monitor milk composition with oil supplementation; cost-effective with current pricing structure; regulatory approval varies by region
Intensive Beef	3-NOP	Nitrate	Include in concentrate portion; 3-NOP at 150-200 mg/kg DMI or nitrate at 2-3% of diet DM	25-35% reduction	Implement adaptation protocol for nitrate; higher efficacy than in dairy systems; improved feed efficiency may offset costs
Pasture Dairy	Oils	Ionophores	Deliver through supplemental concentrate during milking; oils	8-15% reduction	Intake variability is major limitation; consider self-limiting supplements;

			at 4-6% of supplement DM or label-approved ionophore dose		regulatory restrictions for ionophores vary by region
Pasture Beef	Oils + Phytochemicals	Nitrate	Incorporate in protein/energy supplements or mineral blocks; use combination approach where possible	10-20% reduction	Adaptation period critical for nitrate; supplement intake monitoring recommended; cost-benefit favorable when combined with production benefits
Smallholder Mixed	Oils (locally available)	Phytochemicals (native plants)	Integrate with existing supplementation practices; emphasize dual-purpose interventions with production benefits	10-15% reduction	Focus on locally available resources; prioritize interventions with positive or neutral production effects; implementation simplicity critical
Specialized Applications	Macroalgae	3-NOP + Macroalgae	Target for high-value animals or premium markets; incorporate in specialized feeding systems with precise delivery	40-70% reduction	Currently cost-prohibitive for widespread use; may be justified for carbon credit markets or premium products; requires specialized handling

3. Supplementary Figures

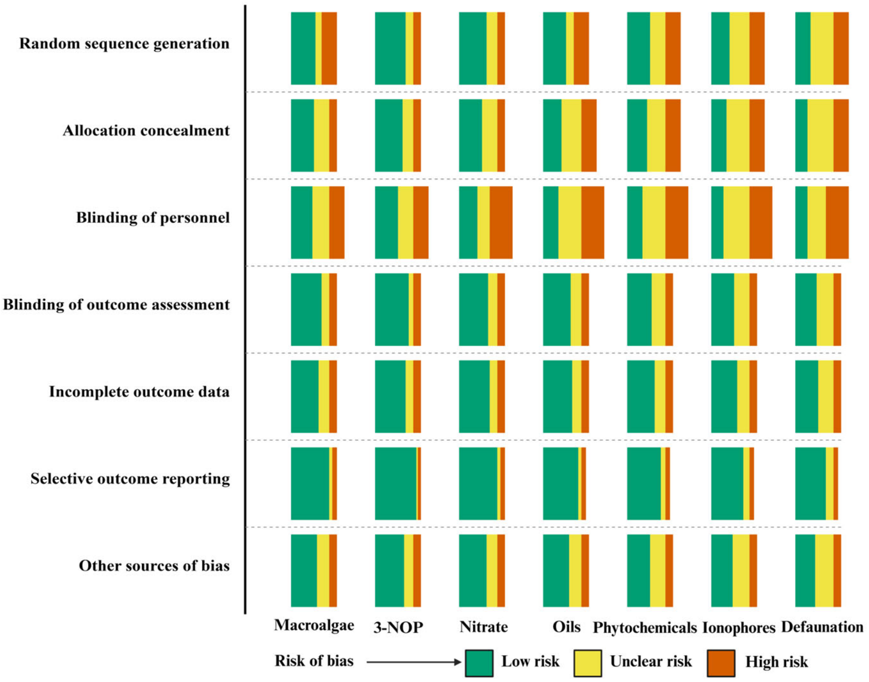

Figure S1. *SYRCLE risk of bias assessment for included studies. The quality assessment results across seven bias domains for all intervention categories, highlighting the methodological rigor variation across studies with color-coded risk levels.*

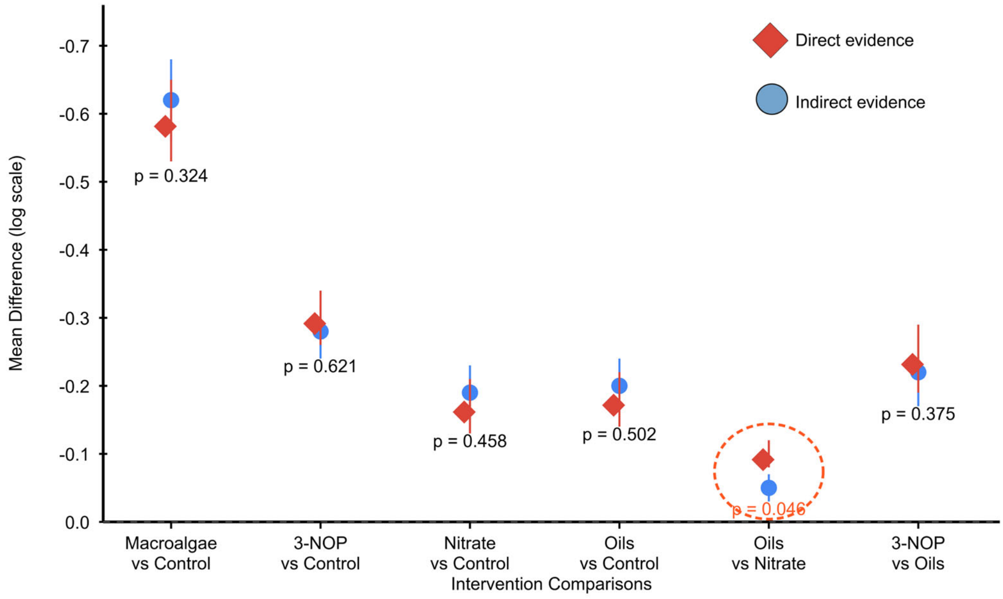

Figure S2. *Network inconsistency analysis. The consistency between direct and indirect evidence for key intervention comparisons, with highlighted inconsistency for oils vs. nitrate comparison.*

References

1. Meo-Filho, P.; Ramirez-Agudelo, J.F.; Kebreab, E. Mitigating methane emissions in grazing beef cattle with a seaweed-based feed additive: Implications for climate-smart agriculture. *Proc. Natl. Acad. Sci. USA* **2024**, *121*, e2410863121. https://doi.org/10.1073/pnas.2410863121.
2. Benchaar, C. Diet supplementation with cinnamon oil, cinnamaldehyde, or monensin does not reduce enteric methane production of dairy cows. *Animal* **2016**, *10*, 418–425. https://doi.org/10.1017/S175173111500230X.
3. Angellotti, M.; Lindberg, M.; Ramin, M.; Krizsan, S.J.; Danielsson, R. Asparagopsis taxiformis supplementation to mitigate enteric methane emissions in dairy cows—Effects on performance and metabolism. *J. Dairy Sci.* **2024**, *108*, 2503–2516. https://doi.org/10.3168/jds.2024-25258.
4. Muizelaar, W.; van Duinkerken, G.; Khan, Z.; Dijkstra, J. Evaluation of 3 northwest European seaweed species on enteric methane production and lactational performance of Holstein-Friesian dairy cows. *J. Dairy Sci.* **2023**, *106*, 4622–4633. https://doi.org/10.3168/jds.2022-22749.
5. Eikanger, K.S.; Kjær, S.T.; Dörsch, P.; Iwaasa, A.D.; Alemu, A.W.; Schei, I.; Pope, P.B.; Hagen, L.H.; Kidane, A. *Asparagopsis taxiformis* inclusion in grass silage-based diets fed to Norwegian red dairy cows: Effects on ruminal fermentation, milk yield, and enteric methane emission. *Livest. Sci.* **2024**, *285*, 105495. https://doi.org/10.1016/j.livsci.2024.105495.
6. Roque, B.M.; Venegas, M.; Kinley, R.D.; de Nys, R.; Duarte, T.L.; Yang, X.; Kebreab, E. Red seaweed (Asparagopsis taxiformis) supplementation reduces enteric methane by over 80 percent in beef steers. *PLoS ONE* **2021**, *16*, e0247820. https://doi.org/10.1371/journal.pone.0247820.
7. Hristov, A.N.; Lee, C.; Cassidy, T.; Heyler, K.; Tekippe, J.A.; Varga, G.A.; Corl, B.; Brandt, R.C. Effect of *Origanum vulgare* L. leaves on rumen fermentation, production, and milk fatty acid composition in lactating dairy cows. *J. Dairy Sci.* **2013**, *96*, 1189–1202. https://doi.org/10.3168/jds.2012-5975.
8. Magnani, E.; Silva, T.H.; Sakamoto, L.; Manella, M.Q.; Dias, F.M.G.N.; Mercadante, M.E.; Henry, D.; Marcatto, J.O.S.; Paula, E.M.; Branco, R.H. Tannin-based product in feedlot diet as a strategy to reduce enteric methane emissions of Nellore cattle finished under tropical conditions. *Transl. Anim. Sci.* **2023**, *7*, txad048. https://doi.org/10.1093/tas/txad048.
9. Silva, T.H.; Magnani, E.; de Andrade, J.G.P.; Amâncio, B.R.; Meurer, G.W.; Reolon, H.G.; Benedeti, P.D.B.; Paula, E.M.; Branco, R.H. Evaluation of increasing levels of condensed tannin extracted from Acacia mearnsii on performance, carcass traits, meat quality, methane emission, and health of finishing Nellore bulls. *Anim. Feed Sci. Technol.* **2024**, *315*, 116046. https://doi.org/10.1016/j.anifeedsci.2024.116046.
10. Teobaldo, R.W.; Cardoso, A.D.; Brito, T.R.; Leite, R.G.; Romanzini, E.P.; Granja-Salcedo, Y.T.; Reis, R.A. Response of phytogenic additives on enteric methane emissions and animal performance of nellore bulls raised in grassland. *Sustainability* **2022**, *14*, 9395.
11. Stefenoni, H.A.; Räisänen, S.E.; Cueva, S.F.; Wasson, D.E.; Lage, C.F.A.; Melgar, A.; Fetter, M.E.; Smith, P.; Hennessy, M.; Vecchiarelli, B.; et al. Effects of the macroalga Asparagopsis taxiformis and oregano leaves on methane emission, rumen fermentation, and lactational performance of dairy cows. *J. Dairy Sci.* **2021**, *104*, 4157–4173.
12. Kirwan, S.F.; Tamassia, L.F.M.; Walker, N.D.; Karagiannis, A.; Kindermann, M.; Waters, S.M. Effects of dietary supplementation with 3-nitrooxypropanol on enteric methane production, rumen fermentation, and performance in young growing beef cattle offered a 50:50 forage:concentrate diet. *J. Anim. Sci.* **2024**, *102*, skad399. https://doi.org/10.1093/jas/skad399.
13. Souza, W.L.; Niehues, M.B.; Cardoso, A.D.; Carvalho, V.V.; Perdigão, A.; Acedo, T.S.; Costa, D.F.; Tamassia, L.F.; Kindermann, M.; Reis, R.A. Effect of 3-nitrooxypropanol combined with different feed additives on growth performance, carcass traits, enteric methane emissions, and physiological responses in feedlot beef cattle fed a high-concentrate finishing diet. *Animals* **2024**, *14*, 3488.
14. Machmüller, A.; Ossowski, D.A.; Kreuzer, M. Comparative evaluation of the effects of coconut oil, oilseeds and crystalline fat on methane release, digestion and energy balance in lambs. *Anim. Feed Sci. Technol.* **2000**, *85*, 41–60.
15. Machmüller, A.; Dohme, F.; Soliva, C.R.; Wanner, M.; Kreuzer, M. Diet composition affects the level of ruminal methane suppression by medium-chain fatty acids. *Aust. J. Agric. Res.* **2001**, *52*, 713–722.
16. Beauchemin, K.A.; McGinn, S.M. Methane emissions from beef cattle: Effects of fumaric acid, essential oil, and canola oil. *J. Anim. Sci.* **2006**, *84*, 1489–1496. https://doi.org/10.2527/2006.8461489x.
17. Beauchemin, K.A.; McGinn, S.M.; Petit, H.V. Methane abatement strategies for cattle: Lipid supplementation of diets. *Can. J. Anim. Sci.* **2007**, *87*, 431–440. https://doi.org/10.4141/cjas07011.
18. Beauchemin, K.A.; McGinn, S.M.; Benchaar, C.; Holtshausen, L. Crushed sunflower, flax, or canola seeds in lactating dairy cow diets: Effects on methane production, rumen fermentation, and milk production. *J. Dairy Sci.* **2009**, *92*, 2118–2127. https://doi.org/10.3168/jds.2008-1903.

19. Hess, H.D.; Beuret, R.A.; Lötscher, M.; Hindrichsen, I.K.; Machmüller, A.; Carulla, J.E.; Lascano, C.E.; Kreuzer, M. Ruminal fermentation, methanogenesis and nitrogen utilization of sheep receiving tropical grass hay-concentrate diets offered with *Sapindus saponaria* fruits and *Cratylia argentea* foliage. *Anim. Sci.* **2016**, *79*, 177–189. https://doi.org/10.1017/S1357729800054643.
20. Benchaar, C.; Hassanat, F.; Martineau, R.; Gervais, R. Linseed oil supplementation to dairy cows fed diets based on red clover silage or corn silage: Effects on methane production, rumen fermentation, nutrient digestibility, N balance, and milk production. *J. Dairy Sci.* **2015**, *98*, 7993–8008. https://doi.org/10.3168/jds.2015-9398.
21. Bird, S.H.; Hegarty, R.S.; Woodgate, R. Persistence of defaunation effects on digestion and methane production in ewes. *Aust. J. Agric. Res.* **2008**, *48*, 152–155.
22. Caetano, M.; Wilkes, M.J.; Pitchford, W.S.; Lee, S.J.; Hynd, P.I. Effect of ensiled crimped grape marc on energy intake, performance and gas emissions of beef cattle. *Anim. Feed Sci. Technol.* **2019**, *247*, 166–172. https://doi.org/10.1016/j.anifeedsci.2018.10.007.
23. Carulla, J.E.; Kreuzer, M.; Machmüller, A.; Hess, H.D. Supplementation of *Acacia mearnsii* tannins decreases methanogenesis and urinary nitrogen in forage-fed sheep. *Aust. J. Exp. Agric.* **2005**, *56*, 961–970.
24. Carvalho, I.P.C.D.; Fiorentini, G.; Berndt, A.; Castagnino, P.D.S.; Messana, J.D.; Frighetto, R.T.S.; Reis, R.A.; Berchielli, T.T. Performance and methane emissions of Nellore steers grazing tropical pasture supplemented with lipid sources. *Rev. Bras. Zootec.* **2016**, *45*, 760–767.
25. Chung, Y.H.; Walker, N.D.; McGinn, S.M.; Beauchemin, K.A. Differing effects of 2 active dried yeast (*Saccharomyces cerevisiae*) strains on ruminal acidosis and methane production in nonlactating dairy cows. *J. Dairy Sci.* **2011**, *94*, 2431–2439. https://doi.org/10.3168/jds.2010-3277.
26. Cooprider, K.L.; Mitloehner, F.M.; Famula, T.R.; Kebreab, E.; Zhao, Y.; Van Eenennaam, A.L. Feedlot efficiency implications on greenhouse gas emissions and sustainability. *J. Anim. Sci.* **2011**, *89*, 2643–2656. https://doi.org/10.2527/jas.2010-3539.
27. Cosgrove, G.P.; Waghorn, G.C.; Anderson, C.B.; Peters, J.S.; Smith, A.; Molano, G.; Deighton, M. The effect of oils fed to sheep on methane production and digestion of ryegrass pasture. *Aust. J. Agric. Res.* **2008**, *48*, 189–192.
28. Ding, X.; Long, R.; Zhang, Q.; Huang, X.; Guo, X.; Mi, J. Reducing methane emissions and the methanogen population in the rumen of Tibetan sheep by dietary supplementation with coconut oil. *Trop. Anim. Health Prod.* **2012**, *44*, 1541–1545. https://doi.org/10.1007/s11250-012-0103-7.
29. Duthie, C.A.; Troy, S.M.; Hyslop, J.J.; Ross, D.W.; Roehe, R.; Rooke, J.A. The effect of dietary addition of nitrate or increase in lipid concentrations, alone or in combination, on performance and methane emissions of beef cattle. *Animal* **2018**, *12*, 280–287. https://doi.org/10.1017/S175173111700146X.
30. El-Zaiat, H.M.; Araujo, R.C.; Soltan, Y.A.; Morsy, A.S.; Louvandini, H.; Pires, A.V.; Patino, H.O.; Correa, P.S.; Abdalla, A.L. Encapsulated nitrate and cashew nut shell liquid on blood and rumen constituents, methane emission, and growth performance of lambs. *J. Anim. Sci.* **2014**, *92*, 2214–2224. https://doi.org/10.2527/jas.2013-7084.
31. Fiorentini, G.; Carvalho, I.P.C.; Messana, J.D.; Castagnino, P.S.; Berndt, A.; Canesin, R.C.; Frighetto, R.T.S.; Berchielli, T.T. Effect of lipid sources with different fatty acid profiles on the intake, performance, and methane emissions of feedlot Nellore steers. *J. Anim. Sci.* **2014**, *92*, 1613–1620. https://doi.org/10.2527/jas.2013-6868.
32. Alemu, A.W.; Romero-Perez, A.; Araujo, R.C.; Beauchemin, K.A. Effect of encapsulated nitrate and microencapsulated blend of essential oils on growth performance and methane emissions from beef steers fed backgrounding diets. *Animals* **2019**, *9*, 21. https://doi.org/10.3390/ani9010021.
33. Grainger, C.; Clarke, T.; Beauchemin, K.A.; McGinn, S.M.; Eckard, R.J. Supplementation with whole cottonseed reduces methane emissions and can profitably increase milk production of dairy cows offered a forage and cereal grain diet. *Aust. J. Agric. Res.* **2008**, *48*, 73–76.
34. Grainger, C.; Auldist, M.J.; Clarke, T.; Beauchemin, K.A.; McGinn, S.M.; Hannah, M.C.; Eckard, R.J.; Lowe, L.B. Use of monensin controlled-release capsules to reduce methane emissions and improve milk production of dairy cows offered pasture supplemented with grain. *J. Dairy Sci.* **2008**, *91*, 1159–1165. https://doi.org/10.3168/jds.2007-0319.
35. Grainger, C.; Clarke, T.; Auldist, M.J.; Beauchemin, K.A.; McGinn, S.M.; Waghorn, G.C.; Eckard, R.J. Potential use of *Acacia mearnsii* condensed tannins to reduce methane emissions and nitrogen excretion from grazing dairy cows. *Can. J. Anim. Sci.* **2009**, *89*, 241–251. https://doi.org/10.4141/CJAS08110.
36. Grainger, C.; Williams, R.; Eckard, R.J.; Hannah, M.C. A high dose of monensin does not reduce methane emissions of dairy cows offered pasture supplemented with grain. *J. Dairy Sci.* **2010**, *93*, 5300–5308. https://doi.org/10.3168/jds.2010-3154.

37. Granja-Salcedo, Y.T.; Fernandes, R.M.; Araujo, R.C.D.; Kishi, L.T.; Berchielli, T.T.; Resende, F.D.D.; Berndt, A.; Siqueira, G.R. Long-term encapsulated nitrate supplementation modulates rumen microbial diversity and rumen fermentation to reduce methane emission in grazing steers. *Front. Microbiol.* **2019**, *10*, 614.
38. Guyader, J.; Eugène, M.; Doreau, M.; Morgavi, D.P.; Gérard, C.; Loncke, C.; Martin, C. Nitrate but not tea saponin feed additives decreased enteric methane emissions in nonlactating cows. *J. Anim. Sci.* **2015**, *93*, 5367–5377. https://doi.org/10.2527/jas.2015-9367.
39. Guyader, J.; Eugène, M.; Meunier, B.; Doreau, M.; Morgavi, D.P.; Silberberg, M.; Rochette, Y.; Gerard, C.; Loncke, C.; Martin, C. Additive methane-mitigating effect between linseed oil and nitrate fed to cattle. *J. Anim. Sci.* **2015**, *93*, 3564–3577. https://doi.org/10.2527/jas.2014-8196.
40. Guyader, J.; Doreau, M.; Morgavi, D.P.; Gérard, C.; Loncke, C.; Martin, C. Long-term effect of linseed plus nitrate fed to dairy cows on enteric methane emission and nitrate and nitrite residuals in milk. *Animal* **2016**, *10*, 1173–1181. https://doi.org/10.1017/S1751731115002852.
41. Haisan, J.; Sun, Y.; Guan, L.L.; Beauchemin, K.A.; Iwaasa, A.; Duval, S.; Barreda, D.R.; Oba, M. The effects of feeding 3-nitrooxypropanol on methane emissions and productivity of Holstein cows in mid lactation. *J. Dairy Sci.* **2014**, *97*, 3110–3119. https://doi.org/10.3168/jds.2013-7834.
42. Haisan, J.; Sun, Y.; Guan, L.; Beauchemin, K.A.; Iwaasa, A.; Duval, S.; Kindermann, M.; Barreda, D.R.; Oba, M. The effects of feeding 3-nitrooxypropanol at two doses on milk production, rumen fermentation, plasma metabolites, nutrient digestibility, and methane emissions in lactating Holstein cows. *Anim. Prod. Sci.* **2017**, *57*, 282–289. https://doi.org/10.1071/an15219.
43. Hegarty, R.S.; Bird, S.H.; Vanselow, B.A.; Woodgate, R. Effects of the absence of protozoa from birth or from weaning on the growth and methane production of lambs. *Brit. J. Nutr.* **2008**, *100*, 1220–1227. https://doi.org/10.1017/S0007114508981435.
44. Hollmann, M.; Powers, W.J.; Fogiel, A.C.; Liesman, J.S.; Bello, N.M.; Beede, D.K. Enteric methane emissions and lactational performance of Holstein cows fed different concentrations of coconut oil. *J. Dairy Sci.* **2012**, *95*, 2602–2615. https://doi.org/10.3168/jds.2011-4896.
45. Holtshausen, L.; Chaves, A.V.; Beauchemin, K.A.; McGinn, S.M.; McAllister, T.A.; Odongo, N.E.; Cheeke, P.R.; Benchaar, C. Feeding saponin-containing *Yucca schidigera* and *Quillaja saponaria* to decrease enteric methane production in dairy cows1. *J. Dairy Sci.* **2009**, *92*, 2809–2821. https://doi.org/10.3168/jds.2008-1843.
46. Hosoda, K.; Nishida, T.; Park, W.Y.; Eruden, B. Influence of *mentha × piperita* l. (peppermint) supplementation on nutrient digestibility and energy metabolism in lactating dairy cows. *Asian-Australas J. Anim. Sci.* **2005**, *18*, 1721–1726. https://doi.org/10.5713/ajas.2005.1721.
47. Hristov, A.N.; Oh, J.; Giallongo, F.; Frederick, T.W.; Harper, M.T.; Weeks, H.L.; Branco, A.F.; Moate, P.J.; Deighton, M.H.; Williams, S.R.; et al. An inhibitor persistently decreased enteric methane emission from dairy cows with no negative effect on milk production. *Proc. Natl. Acad. Sci. USA* **2015**, *112*, 10663–10668. https://doi.org/10.1073/pnas.1504124112.
48. Hulshof, R.B.; Berndt, A.; Gerrits, W.J.; Dijkstra, J.; van Zijderveld, S.M.; Newbold, J.R.; Perdok, H.B. Dietary nitrate supplementation reduces methane emission in beef cattle fed sugarcane-based diets. *J. Anim. Sci.* **2012**, *90*, 2317–2323. https://doi.org/10.2527/jas.2011-4209.
49. Hünerberg, M.; McGinn, S.M.; Beauchemin, K.A.; Okine, E.K.; Harstad, O.M.; McAllister, T.A. Effect of dried distillers grains plus solubles on enteric methane emissions and nitrogen excretion from growing beef cattle1. *J. Anim. Sci.* **2013**, *91*, 2846–2857. https://doi.org/10.2527/jas.2012-5564.
50. Hünerberg, M.; McGinn, S.M.; Beauchemin, K.A.; Okine, E.K.; Harstad, O.M.; McAllister, T.A. Effect of dried distillers' grains with solubles on enteric methane emissions and nitrogen excretion from finishing beef cattle. *Can. J. Anim. Sci.* **2013**, *93*, 373–385. https://doi.org/10.4141/cjas2012-151.
51. Johnson, K.A.; Kincaid, R.L.; Westberg, H.H.; Gaskins, C.T.; Lamb, B.K.; Cronrath, J.D. The effect of oilseeds in diets of lactating cows on milk production and methane emissions. *J. Dairy Sci.* **2002**, *85*, 1509–1515. https://doi.org/10.3168/jds.S0022-0302(02)74220-3.
52. Jordan, E.; Kenny, D.; Hawkins, M.; Malone, R.; Lovett, D.K.; O'Mara, F.P. Effect of refined soy oil or whole soybeans on intake, methane output, and performance of young bulls. *J. Anim. Sci.* **2006**, *84*, 2418–2425. https://doi.org/10.2527/jas.2005-354.
53. Jordan, E.; Lovett, D.K.; Hawkins, M.; Callan, J.J.; O'Mara, F.P. The effect of varying levels of coconut oil on intake, digestibility and methane output from continental cross beef heifers. *Anim. Sci.* **2006**, *82*, 859–865. https://doi.org/10.1017/ASC2006107.
54. Jordan, E.; Lovett, D.K.; Monahan, F.J.; Callan, J.; Flynn, B.; O'Mara, F.P. Effect of refined coconut oil or copra meal on methane output and on intake and performance of beef heifers. *J. Anim. Sci.* **2006**, *84*, 162–170. https://doi.org/10.2527/2006.841162x.

55. Neto, A.J.; Messana, J.D.; Rossi, L.G.; Carvalho, I.P.C.; Berchielli, T.T. Methane emissions from Nellore bulls on pasture fed two levels of starch-based supplement with or without a source of oil. *Anim. Prod. Sci.* **2018**, *59*, 654–663.
56. Kim, S.A.; Lee, C.; Pechtl, H.A.; Hettick, J.M.; Campler, M.R.; Pairis-Garcia, M.D.; Beauchemin, K.A.; Celi, P.; Duval, S.M. Effects of 3-nitrooxypropanol on enteric methane production, rumen fermentation, and feeding behavior in beef cattle fed a high-forage or high-grain diet. *J. Anim. Sci.* **2019**, *97*, 2687–2699. https://doi.org/10.1093/jas/skz140.
57. Kinley, R.D.; Martinez-Fernandez, G.; Matthews, M.K.; de Nys, R.; Magnusson, M.; Tomkins, N.W. Mitigating the carbon footprint and improving productivity of ruminant livestock agriculture using a red seaweed. *J. Clean. Prod.* **2020**, *259*, 120836. https://doi.org/10.1016/j.jclepro.2020.120836.
58. Klevenhusen, F.; Zeitz, J.O.; Duval, S.; Kreuzer, M.; Soliva, C.R. Garlic oil and its principal component diallyl disulfide fail to mitigate methane, but improve digestibility in sheep. *Anim. Feed Sci. Technol.* **2011**, *166–167*, 356–363. https://doi.org/10.1016/j.anifeedsci.2011.04.071.
59. Lee, C.; Araujo, R.C.; Koenig, K.M.; Beauchemin, K.A. Effects of encapsulated nitrate on enteric methane production and nitrogen and energy utilization in beef heifers. *J. Anim. Sci.* **2015**, *93*, 2391–2404. https://doi.org/10.2527/jas.2014-8845.
60. Lee, C.; Araujo, R.C.; Koenig, K.M.; Beauchemin, K.A. Effects of encapsulated nitrate on growth performance, carcass characteristics, nitrate residues in tissues, and enteric methane emissions in beef steers: Finishing phase. *J. Anim. Sci.* **2017**, *95*, 3712–3726. https://doi.org/10.2527/jas.2017.1461.
61. Li, L.; Davis, J.; Nolan, J.; Hegarty, R. An initial investigation on rumen fermentation pattern and methane emission of sheep offered diets containing urea or nitrate as the nitrogen source. *Anim. Prod. Sci.* **2012**, *52*, 653–658.
62. Li, L.; Silveira, C.I.; Nolan, J.V.; Godwin, I.R.; Leng, R.A.; Hegarty, R.S. Effect of added dietary nitrate and elemental sulfur on wool growth and methane emission of Merino lambs. *Anim. Prod. Sci.* **2013**, *53*, 1195–1201.
63. Li, X.; Norman, H.C.; Kinley, R.D.; Laurence, M.; Wilmot, M.; Bender, H.; de Nys, R.; Tomkins, N. *Asparagopsis taxiformis* decreases enteric methane production from sheep. *Anim. Prod. Sci.* **2018**, *58*, 681–688.
64. Liu, H.; Vaddella, V.; Zhou, D. Effects of chestnut tannins and coconut oil on growth performance, methane emission, ruminal fermentation, and microbial populations in sheep. *J. Dairy Sci.* **2011**, *94*, 6069–6077. https://doi.org/10.3168/jds.2011-4508.
65. Lopes, J.C.; de Matos, L.F.; Harper, M.T.; Giallongo, F.; Oh, J.; Gruen, D.; Ono, S.; Kindermann, M.; Duval, S.; Hristov, A.N. Effect of 3-nitrooxypropanol on methane and hydrogen emissions, methane isotopic signature, and ruminal fermentation in dairy cows. *J. Dairy Sci.* **2016**, *99*, 5335–5344. https://doi.org/10.3168/jds.2015-10832.
66. Ma, T.; Chen, D.D.; Tu, Y.; Zhang, N.F.; Si, B.W.; Deng, K.D.; Diao, Q.Y. Effect of dietary supplementation with resveratrol on nutrient digestibility, methanogenesis and ruminal microbial flora in sheep. *J. Anim. Physiol. Anim. Nutr.* **2015**, *99*, 676–683. https://doi.org/10.1111/jpn.12264.
67. Ma, T.; Chen, D.D.; Tu, Y.; Zhang, N.F.; Si, B.W.; Diao, Q.Y. Dietary supplementation with mulberry leaf flavonoids inhibits methanogenesis in sheep. *Anim. Sci. J.* **2016**, *88*, 72–78. https://doi.org/10.1111/asj.12556.
68. Machmüller, A.; Soliva, C.R.; Kreuzer, M. Methane-suppressing effect of myristic acid in sheep as affected by dietary calcium and forage proportion. *Brit. J. Nutr.* **2003**, *90*, 529–540. https://doi.org/10.1079/BJN2003932.
69. Malik, P.K.; Kolte, A.P.; Baruah, L.; Saravanan, M.; Bakshi, B.; Bhatta, R. Enteric methane mitigation in sheep through leaves of selected tanniniferous tropical tree species. *Livest. Sci.* **2017**, *200*, 29–34. https://doi.org/10.1016/j.livsci.2017.04.001.
70. Mao, H.L.; Wang, J.K.; Zhou, Y.Y.; Liu, J.X. Effects of addition of tea saponins and soybean oil on methane production, fermentation and microbial population in the rumen of growing lambs. *Livest. Sci.* **2010**, *129*, 56–62. https://doi.org/10.1016/j.livsci.2009.12.011.
71. Martin, C.; Rouel, J.; Jouany, J.P.; Doreau, M.; Chilliard, Y. Methane output and diet digestibility in response to feeding dairy cows crude linseed, extruded linseed, or linseed oil. *J. Anim. Sci.* **2008**, *86*, 2642–2650. https://doi.org/10.2527/jas.2007-0774.
72. Martin, C.; Ferlay, A.; Mosoni, P.; Rochette, Y.; Chilliard, Y.; Doreau, M. Increasing linseed supply in dairy cow diets based on hay or corn silage: Effect on enteric methane emission, rumen microbial fermentation, and digestion. *J. Dairy Sci.* **2016**, *99*, 3445–3456. https://doi.org/10.3168/jds.2015-10110.
73. Martinez-Fernandez, G.; Duval, S.; Kindermann, M.; Schirra, H.J.; Denman, S.E.; McSweeney, C.S. 3-NOP vs. Halogenated Compound: Methane Production, Ruminal Fermentation and Microbial Community Response in Forage Fed Cattle. *Front. Microbiol.* **2018**, *9*, 1582. https://doi.org/10.3389/fmicb.2018.01582.
74. McGinn, S.M.; Beauchemin, K.A.; Coates, T.; Colombatto, D. Methane emissions from beef cattle: Effects of monensin, sunflower oil, enzymes, yeast, and fumaric acid. *J. Anim. Sci.* **2004**, *82*, 3346–3356. https://doi.org/10.2527/2004.82113346x.
75. McGinn, S.M.; Chung, Y.H.; Beauchemin, K.A.; Iwaasa, A.D.; Grainger, C. Use of corn distillers' dried grains to reduce enteric methane loss from beef cattle. *Can. J. Anim. Sci.* **2009**, *89*, 409–413. https://doi.org/10.4141/CJAS08133.

76. Melgar, A.; Harper, M.T.; Oh, J.; Giallongo, F.; Young, M.E.; Ott, T.L.; Duval, S.; Hristov, A.N. Effects of 3-nitrooxypropanol on rumen fermentation, lactational performance, and resumption of ovarian cyclicity in dairy cows. *J. Dairy Sci.* **2020**, *104*, 3157–3172. https://doi.org/10.3168/jds.2020-19307.
77. Moate, P.J.; Williams, S.R.O.; Grainger, C.; Hannah, M.C.; Ponnampalam, E.N.; Eckard, R.J. Influence of cold-pressed canola, brewers grains and hominy meal as dietary supplements suitable for reducing enteric methane emissions from lactating dairy cows. *Anim. Feed Sci. Technol.* **2011**, *166–167*, 254–264. https://doi.org/10.1016/j.anifeedsci.2011.04.069.
78. Moate, P.J.; Williams, S.R.O.; Torok, V.A.; Hannah, M.C.; Ribaux, B.E.; Tavendale, M.H.; Eckard, R.J.; Jacobs, J.L.; Auldist, M.J.; Wales, W.J. Grape marc reduces methane emissions when fed to dairy cows. *J. Dairy Sci.* **2014**, *97*, 5073–5087. https://doi.org/10.3168/jds.2013-7588.
79. Mohammed, N.; Ajisaka, N.; Lila, Z.A.; Hara, K.; Mikuni, K.; Hara, K.; Kanda, S.; Itabashi, H. Effect of Japanese horseradish oil on methane production and ruminal fermentation in vitro and in steers. *J. Anim. Sci.* **2004**, *82*, 1839–1846. https://doi.org/10.2527/2004.8261839x.
80. Moreira, G.D.; Lima, P.M.T.; Borges, B.O.; Primavesi, O.; Longo, C.; McManus, C.; Abdalla, A.; Louvandini, H. Tropical tanniniferous legumes used as an option to mitigate sheep enteric methane emission. *Trop. Anim. Health Prod.* **2013**, *45*, 879–882. https://doi.org/10.1007/s11250-012-0284-0.
81. Mwenya, B.; Santoso, B.; Sar, C.; Pen, B.; Morikawa, R.; Takaura, K.; Umetsu, K.; Kimura, K.; Takahashi, J. Effects of yeast culture and galacto-oligosaccharides on ruminal fermentation in holstein cows. *J. Dairy Sci.* **2005**, *88*, 1404–1412. https://doi.org/10.3168/jds.S0022-0302(05)72808-3.
82. Newbold, J.R.; van Zijderveld, S.M.; Hulshof, R.B.A.; Fokkink, W.B.; Leng, R.A.; Terencio, P.; Powers, W.J.; van Adrichem, P.S.J.; Paton, N.D.; Perdok, H.B. The effect of incremental levels of dietary nitrate on methane emissions in Holstein steers and performance in Nelore bulls. *J. Anim. Sci.* **2014**, *92*, 5032–5040. https://doi.org/10.2527/jas.2014-7677.
83. Nguyen, S.H.; Hegarty, R.S. Effects of defaunation and dietary coconut oil distillate on fermentation, digesta kinetics and methane production of Brahman heifers. *J. Anim. Physiol. Anim. Nutr.* **2017**, *101*, 984–993. https://doi.org/10.1111/jpn.12534.
84. Nolan, J.V.; Hegarty, R.S.; Hegarty, J.; Godwin, I.R.; Woodgate, R. Effects of dietary nitrate on fermentation, methane production and digesta kinetics in sheep. *Anim. Prod. Sci.* **2010**, *50*, 801–806. https://doi.org/10.1071/AN09211.
85. Norris, A.B.; Crossland, W.L.; Tedeschi, L.O.; Foster, J.L.; Muir, J.P.; Pinchak, W.E.; Fonseca, M.A. Inclusion of quebracho tannin extract in a high-roughage cattle diet alters digestibility, nitrogen balance, and energy partitioning. *J. Anim. Sci.* **2020**, *98*, skaa047. https://doi.org/10.1093/jas/skaa047.
86. Odongo, N.E.; Or-Rashid, M.M.; Kebreab, E.; France, J.; McBride, B.W. Effect of supplementing myristic acid in dairy cow rations on ruminal methanogenesis and fatty acid profile in milk. *J. Dairy Sci.* **2007**, *90*, 1851–1858. https://doi.org/10.3168/jds.2006-541.
87. Odongo, N.E.; Bagg, R.; Vessie, G.; Dick, P.; Or-Rashid, M.M.; Hook, S.E.; Gray, J.T.; Kebreab, E.; France, J.; McBride, B.W. Long-term effects of feeding monensin on methane production in lactating dairy cows. *J. Dairy Sci.* **2007**, *90*, 1781–1788. https://doi.org/10.3168/jds.2006-708.
88. Olijhoek, D.W.; Hellwing, A.L.F.; Brask, M.; Weisbjerg, M.R.; Hojberg, O.; Larsen, M.K.; Dijkstra, J.; Erlandsen, E.J.; Lund, P. Effect of dietary nitrate level on enteric methane production, hydrogen emission, rumen fermentation, and nutrient digestibility in dairy cows. *J. Dairy Sci.* **2016**, *99*, 6191–6205. https://doi.org/10.3168/jds.2015-10691.
89. de Oliveira, S.G.; Berchielli, T.T.; Pedreira, M.D.S.; Primavesi, O.; Frighetto, R.; Lima, M.A. Effect of tannin levels in sorghum silage and concentrate supplementation on apparent digestibility and methane emission in beef cattle. *Anim. Feed Sci. Technol.* **2007**, *135*, 236–248. https://doi.org/10.1016/j.anifeedsci.2006.07.012.
90. Patra, A.K.; Kamra, D.N.; Bhar, R.; Kumar, R.; Agarwal, N. Effect of *Terminalia chebula* and Allium sativum on in vivo methane emission by sheep. *J. Anim. Physiol. Anim. Nutr.* **2011**, *95*, 187–191. https://doi.org/10.1111/j.1439-0396.2010.01039.x.
91. Pen, B.; Takaura, K.; Yamaguchi, S.; Asa, R.; Takahashi, J. Effects of *Yucca schidigera* and *Quillaja saponaria* with or without β 1–4 galacto-oligosaccharides on ruminal fermentation, methane production and nitrogen utilization in sheep. *Anim. Feed Sci. Technol.* **2007**, *138*, 75–88. https://doi.org/10.1016/j.anifeedsci.2006.11.018.
92. Rebelo, L.R.; Luna, I.C.; Messana, J.D.; Araujo, R.C.; Simioni, T.A.; Granja-Salcedo, Y.T.; Vito, E.S.; Lee, C.; Teixeira, I.A.M.A.; Rooke, J.A. Effect of replacing soybean meal with urea or encapsulated nitrate with or without elemental sulfur on nitrogen digestion and methane emissions in feedlot cattle. *Anim. Feed Sci. Technol.* **2019**, *257*, 114293.
93. Reynolds, C.K.; Humphries, D.J.; Kirton, P.; Kindermann, M.; Duval, S.; Steinberg, W. Effects of 3-nitrooxypropanol on methane emission, digestion, and energy and nitrogen balance of lactating dairy cows. *J. Dairy Sci.* **2014**, *97*, 3777–3789. https://doi.org/10.3168/jds.2013-7397.

94. Romero-Perez, A.; Okine, E.K.; McGinn, S.M.; Guan, L.L.; Oba, M.; Duval, S.M.; Kindermann, M.; Beauchemin, K.A. The potential of 3-nitrooxypropanol to lower enteric methane emissions from beef cattle. *J. Anim. Sci.* **2014**, *92*, 4682–4693. https://doi.org/10.2527/jas.2014-7573.
95. Romero-Perez, A.; Okine, E.K.; McGinn, S.M.; Guan, L.L.; Oba, M.; Duval, S.M.; Kindermann, M.; Beauchemin, K.A. Sustained reduction in methane production from long-term addition of 3-nitrooxypropanol to a beef cattle diet1. *J. Anim. Sci.* **2015**, *93*, 1780–1791. https://doi.org/10.2527/jas.2014-8726.
96. Roque, B.M.; Salwen, J.K.; Kinley, R.; Kebreab, E. Inclusion of Asparagopsis armata in lactating dairy cows' diet reduces enteric methane emission by over 50 percent. *J. Clean. Prod.* **2019**, *234*, 118196. https://doi.org/10.1016/j.jclepro.2019.118196.
97. Rossi, L.G.; Fiorentini, G.; Vieira, B.R.; Neto, A.J.; Messana, J.D.; Malheiros, E.B.; Berchielli, T.T. Effect of ground soybean and starch on intake, digestibility, performance, and methane production of Nellore bulls. *Anim. Feed Sci. Technol.* **2017**, *226*, 39–47. https://doi.org/10.1016/j.anifeedsci.2017.02.004.
98. Santoso, B.; Mwenya, B.; Sar, C.; Gamo, Y.; Kobayashi, T.; Morikawa, R.; Kimura, K.; Mizukoshi, H.; Takahashi, J. Effects of supplementing galacto-oligosaccharides, Yucca schidigera or nisin on rumen methanogenesis, nitrogen and energy metabolism in sheep. *Livest. Prod. Sci.* **2004**, *91*, 209–217. https://doi.org/10.1016/j.livprodsci.2004.08.004.
99. Silva, R.A.; Fiorentini, G.; Messana, J.D.; Lage, J.F.; Castagnino, P.S.; San Vito, E.; Carvalho, I.P.C.; Berchielli, T.T. Effects of different forms of soybean lipids on enteric methane emission, performance and meat quality of feedlot Nellore. *J. Agric. Sci.* **2018**, *156*, 427–436. https://doi.org/10.1017/s002185961800045x.
100. Soltan, Y.A.; Morsy, A.S.; Sallam, S.M.A.; Ronaldo, C.L.; Louvandini, H.; Kreuzer, M.; Abdalla, A.L. Contribution of condensed tannins and mimosine to the methane mitigation caused by feeding *Leucaena leucocephala*. *Arch. Anim. Nutr.* **2013**, *67*, 169–184. https://doi.org/10.1080/1745039X.2013.801139.
101. Staerfl, S.M.; Zeitz, J.O.; Kreuzer, M.; Soliva, C.R. Methane conversion rate of bulls fattened on grass or maize silage as compared with the IPCC default values, and the long-term methane mitigation efficiency of adding acacia tannin, garlic, maca and lupine. *Agric. Ecosyst. Environ.* **2012**, *148*, 111–120. https://doi.org/10.1016/j.agee.2011.11.003.
102. Sun, Y.K.; Yan, X.G.; Ban, Z.B.; Yang, H.M.; Hegarty, R.S.; Zhao, Y.M. The effect of cysteamine hydrochloride and nitrate supplementation on in-vitro and in-vivo methane production and productivity of cattle. *Anim. Feed Sci. Technol.* **2017**, *232*, 49–56. https://doi.org/10.1016/j.anifeedsci.2017.03.016.
103. Tiemann, T.T.; Lascano, C.E.; Wettstein, H.R.; Mayer, A.C.; Kreuzer, M.; Hess, H.D. Effect of the tropical tannin-rich shrub legumes Calliandra calothyrsus and Flemingia macrophylla on methane emission and nitrogen and energy balance in growing lambs. *Animal* **2008**, *2*, 790–799. https://doi.org/10.1017/S1751731108001791.
104. Troy, S.M.; Duthie, C.A.; Hyslop, J.J.; Roehe, R.; Ross, D.W.; Wallace, R.J.; Waterhouse, A.; Rooke, J.A. Effectiveness of nitrate addition and increased oil content as methane mitigation strategies for beef cattle fed two contrasting basal diets. *J. Anim. Sci.* **2015**, *93*, 1815–1823. https://doi.org/10.2527/jas.2014-8688.
105. Van Wesemael, D.; Vandaele, L.; Ampe, B.; Cattrysse, H.; Duval, S.; Kindermann, M.; Fievez, V.; De Campeneere, S.; Peiren, N. Reducing enteric methane emissions from dairy cattle: Two ways to supplement 3-nitrooxypropanol. *J. Dairy Sci.* **2019**, *102*, 1780–1787. https://doi.org/10.3168/jds.2018-14534.
106. van Zijderveld, S.M.; Gerrits, W.J.J.; Apajalahti, J.A.; Newbold, J.R.; Dijkstra, J.; Leng, R.A.; Perdok, H.B. Nitrate and sulfate: Effective alternative hydrogen sinks for mitigation of ruminal methane production in sheep. *J. Dairy Sci.* **2010**, *93*, 5856–5866. https://doi.org/10.3168/jds.2010-3281.
107. Van Zijderveld, S.M.; Dijkstra, J.; Perdok, H.B.; Newbold, J.R.; Gerrits, W.J.J. Dietary inclusion of diallyl disulfide, yucca powder, calcium fumarate, an extruded linseed product, or medium-chain fatty acids does not affect methane production in lactating dairy cows. *J. Dairy Sci.* **2011**, *94*, 3094–3104. https://doi.org/10.3168/jds.2010-4042.
108. van Zijderveld, S.M.; Gerrits, W.J.; Dijkstra, J.; Newbold, J.R.; Hulshof, R.B.; Perdok, H.B. Persistency of methane mitigation by dietary nitrate supplementation in dairy cows. *J. Dairy Sci.* **2011**, *94*, 4028–4038. https://doi.org/10.3168/jds.2011-4236.
109. Velazco, J.I.; Cottle, D.J.; Hegarty, R.S. Methane emissions and feeding behaviour of feedlot cattle supplemented with nitrate or urea. *Anim. Prod. Sci.* **2014**, *54*, 1737–1740. https://doi.org/10.1071/AN14345.
110. Veneman, J.B.; Muetzel, S.; Hart, K.J.; Faulkner, C.L.; Moorby, J.M.; Perdok, H.B.; Newbold, C.J. Does dietary mitigation of enteric methane production affect rumen function and animal productivity in dairy cows? *PLoS ONE* **2015**, *10*, e0140282. https://doi.org/10.1371/journal.pone.0140282.
111. Villar, M.L.; Hegarty, R.S.; Nolan, J.V.; Godwin, I.R.; McPhee, M. The effect of dietary nitrate and canola oil alone or in combination on fermentation, digesta kinetics and methane emissions from cattle. *Anim. Feed Sci. Technol.* **2019**, *259*, 114294. https://doi.org/10.1016/j.anifeedsci.2019.114294.

112. Vyas, D.; McGinn, S.M.; Duval, S.M.; Kindermann, M.K.; Beauchemin, K.A. Effects of sustained reduction of enteric methane emissions with dietary supplementation of 3-nitrooxypropanol on growth performance of growing and finishing beef cattle. *J. Anim. Sci.* **2016**, *94*, 2042–2052. https://doi.org/10.2527/jas.2015-0268.
113. Vyas, D.; McGinn, S.M.; Duval, S.M.; Kindermann, M.K.; Beauchemin, K.A. Optimal dose of 3-nitrooxypropanol for decreasing enteric methane emissions from beef cattle fed high-forage and high-grain diets. *Anim. Prod. Sci.* **2018**, *58*, 1049–1055. https://doi.org/10.1071/an15705.
114. Vyas, D.; Alemu, A.W.; McGinn, S.M.; Duval, S.M.; Kindermann, M.; Beauchemin, K.A. The combined effects of supplementing monensin and 3-nitrooxypropanol on methane emissions, growth rate, and feed conversion efficiency in beef cattle fed high-forage and high-grain diets. *J. Anim. Sci.* **2018**, *96*, 2923–2938. https://doi.org/10.1093/jas/sky174.
115. Waghorn, G.C.; Clark, H.; Taufa, V.; Cavanagh, A. Monensin controlled-release capsules for methane mitigation in pasture-fed dairy cows. *Aust. J. Exp. Agric.* **2008**, *48*, 65–68. https://doi.org/10.1071/EA07299.
116. Wang, C.J.; Wang, S.P.; Zhou, H. Influences of flavomycin, ropadiar, and saponin on nutrient digestibility, rumen fermentation, and methane emission from sheep. *Anim. Feed Sci. Technol.* **2009**, *148*, 157–166. https://doi.org/10.1016/j.anifeedsci.2008.03.008.
117. Yang, K.; Wei, C.; Zhao, G.Y.; Xu, Z.W.; Lin, S.X. Effects of dietary supplementing tannic acid in the ration of beef cattle on rumen fermentation, methane emission, microbial flora and nutrient digestibility. *J. Anim. Physiol. Anim. Nutr.* **2017**, *101*, 302–310. https://doi.org/10.1111/jpn.12531.
118. Zhou, Y.Y.; Mao, H.L.; Jiang, F.; Wang, J.K.; Liu, J.X.; McSweeney, C.S. Inhibition of rumen methanogenesis by tea saponins with reference to fermentation pattern and microbial communities in Hu sheep. *Anim. Feed Sci. Technol.* **2011**, *166–167*, 93–100. https://doi.org/10.1016/j.anifeedsci.2011.04.007.
119. Vázquez-Carrillo, M.F.; Montelongo-Pérez, H.D.; González-Ronquillo, M.; Castillo-Gallegos, E.; Castelán-Ortega, O.A. Effects of three herbs on methane emissions from beef cattle. *Animals* **2020**, *10*, 1671.
